# A Case of Hypotonia-Cystinuria Syndrome With Genito-Urinary Malformations and Extrarenal Involvement

**DOI:** 10.3389/fped.2019.00127

**Published:** 2019-04-09

**Authors:** Francesca Taroni, Valentina Capone, Alfredo Berrettini, Erika Adalgisa De Marco, Gian Antonio Manzoni, Giovanni Montini

**Affiliations:** ^1^Pediatric Nephrology, Dialysis and Transplant Unit, Fondazione IRCCS Ca' Granda—Ospedale Maggiore Policlinico, Milan, Italy; ^2^Pediatric Urology Unit, Fondazione IRCCS Ca' Granda—Ospedale Maggiore Policlinico, Milan, Italy; ^3^Giuliana and Bernardo Caprotti Chair of Pediatrics, Department of Clinical Sciences and Community Health, University of Milan, Milan, Italy

**Keywords:** hypotonia cystinuria syndrome, PREPL, SLC3A1, CAKUT, patent foramen ovale, cryptorchidism

## Abstract

Hypotonia-Cystinuria syndrome (HCS) is a rare disease, caused by a mutation in two contiguous genes (SLC3A1 and PREPL) localized on chromosome 2p21, and it is characterized by both renal involvement with cystine stones and nervous involvement with hypotonia. We here describe a 2 years old child with HCS associated with other clinical features as congenital anomalies of kidney and urinary tract (primary obstructed megaureter, POM), cryptorchidism and cardiac involvement (patent foramen ovale with atrial septum aneurysm). To the best of our knowledge, cryporchidism and POM have never been reported before in patients with HCS. Moreover, a cardiac involvement has been described only in another case of HCS that, interestingly, presents the same genetic abnormalities as our patient. The diagnosis of HCS can be difficult because neurological signs are aspecific and kidney stones are commonly absent during the first months of life. A better understanding of the complete clinical scenario associated with HCS can help clinicians suspect, diagnose and treat HCS earlier with a positive influence on both neurological and renal outcome.

## Introduction

Hypotonia Cystinuria Syndrome (HCS) is a rare disease, with an estimated prevalence of 1/1.000.000 (1). It is caused by a mutation in two contiguous genes (SLC3A1 and PREPL) localized on chromosome 2p21, and is characterized by both renal involvement with cystine stones and nervous system involvement with hypotonia. To date, only 26 cases of HCS are reported in literature and 8 different deletions have been described.

Cystinuria is an autosomal recessive metabolic disease (with a worldwide prevalence of 1:7.000 newborns) causing urolithiasis, due to mutations in SLC3A1 (Chr 2p21, cystinuria type A) or SLC7A9 (Chr 19q13.11, cystinuria type B). Both genes are expressed in the renal proximal tubule and in the intestine and encode for different subunits of the transporter of dibasic amino acids (cystine, ornithine, lysine, and arginine) (2). Such transporters deficiency leads to an accumulation of cystine in the urinary tract and to subsequent recurrent stones production, which can eventually lead to end stage renal disease. The diagnosis of cystinuria is made when typical flat hexagonal cystine crystals are found in urines or when high levels of urinary cystine (over 300–400 mg/L) are detected. Specific genetic tests allow for the identification of different types of cystinuria. Early diagnosis is crucial because treatment with tiopronin can prevent stone formation and chronic kidney disease (3).

PREPL, or prolyl endopeptidase-like gene, encodes for a protein which belongs to the prolyl oligopeptidase subfamily of serine peptidases, whose function seems to be related to the synaptic vesicle exocytosis. It is expressed ubiquitously, with highest expression in brain, muscle, heart and kidney. Homozygous deletions of PREPL are associated with generalized hypotonia similar to Prader-Willi Syndrome. Hypotonia caused by PREPL deletions can benefit from an early treatment with pyridostigmine (4).

Diagnosis of HCS can be difficult because neurological signs are aspecific and kidney stones are commonly absent during the first months of life. The addition of atypical and novel clinical cases can lead to a better understanding of the genetic and clinical basis of the syndrome, and avoid delayed diagnosis and treatment.

We here report a case of HCS with an atypical presentation due to the association with cardiac and genito-urinary involvement.

## Case Report

We report the case of a 2 years old child of healthy, non-consanguineous, Italian parents. All clinical features and diagnostic work-ups at birth are summarized in Table 1.

**Table 1:** Clinical features and diagnostic work-up at birth.

**Prenatal history**	Reduction of fetal movements Postnatal growth failure
**Clinical Examination**	Generalized hypotonia, failure to thrive. Bitemporal narrowing, frontal bossing, dolichocephaly, micrognathia bilateral ptosis. Left cryptorchidism.
**METABOLIC WORK-UP**	
**Plasmatic amino acids** **Urinary organic acids** **Ammonium** **Lactate** **Acid/base status**	Normal for age
**GENETIC TESTS**	
**Karyotype** **CGH array** **Prader Willi Syndrome**	46, XY Normal No deletion on chromosome 15
**NEUROLOGIC TESTS**	
**Cerebral ultrasound** **Spinal and cerebral MRI** **EMG**	Normal
**Cardiologic examination**	Patent *foramen ovale* (POF, 1 cm) with left-right shunt and atrial septum aneurysm.
**Abdominal ultrasound**	Dilatation of the left renal pelvis and ureter (POM), with an antero-posterior pelvic diameter of 0,8 cm, a ureteric diameter at bladder junction of 0,7 cm and normal renal parenchyma and bladder.

Written informed consent was obtained from the participant for the publication of the case report. Neither urolithiasis nor neurological/renal diseases were reported in family history. The pregnancy was characterized by a reduction in fetal movements from the 25th week of gestation. Fetal growth (expressed as weight, length and cranial circumference) was on the 25th percentile during the whole pregnancy. Fetal ultrasound at 20 weeks showed left calyceal dilatation (6 mm) and ipsilateral megaureter (8 mm); renal parenchyma and bladder were normal in thickness and appearance. Amniotic fluid volume was always adequate.

The child was born at full-term (gestational age 38 weeks, birth weight 3,350 g, length 51 cm, head circumference 36.8 cm) by iterative cesarean delivery. At birth, he presented generalized hypotonia, central cyanosis and bradycardia (APGAR score 5-8-10 at I-V-X minute). Central cyanosis and bradycardia improved with mask ventilation for 2 min while hypotonia persisted. During the following 24 h a persistent generalized hypotonia was observed, associated with failure to thrive. Aspecific dysmorphisms with mild bitemporal narrowing and frontal bossing, dolichocephaly, micrognathia and a slight ptosis were also present.

A complete neurologic, metabolic and genetic evaluation was performed during the first days of life (cerebral ultrasound—US, spinal and cerebral MRI, EMG, *fundus oculi*, audiometric test, dosage of plasmatic amino acids, urinary organic acids, ammonium, lactate, very long chain fatty acids and acid/base status assessment, renal end hepatic function, karyotype and CGH array, genetic test for Prader Willi Syndrome). All these tests didn't show any specific alteration responsible for persistent hypotonia.

A cardiologic examination showed the presence of a patent *foramen ovale* (POF, 1 cm) with left-right shunt and atrial septum aneurysm.

The abdominal ultrasound (US) performed at 3 days of life confirmed a dilatation of the left renal pelvis and ureter (POM), with an antero-posterior pelvic diameter of 0,8 cm, a ureteric diameter at bladder junction of 0,7 cm and normal renal parenchyma and bladder.

Renal US was repeated every 6 months and showed a worsening of the left hydro-ureteronephrosis, with a distal and proximal ureter reaching a diameter of 1.4 and 1 cm, respectively at 18 months. A staghorn stone in the left pelvis (14 × 15 mm) and a stone in the upper ipsilateral ureter (7 × 16 mm) were detected. Moreover, a left palpable undescended testis was detected.

At that time the child presented with also growth retardation with weight under the 3rd percentile and length on the 10th percentile with the need of oro-gastric tube to allow nutrition. A global developmental delay of all the motor and social skills and of the speech was also evident. Neurologic examination revealed truncal hypotonia.

The child then underwent a cystoscopic examination with retrograde pyelography that showed a dilated and tortuous megaureter due to a severe obstruction of the uretero-vesical junction that was surgically corrected: it was not possible to cross the stenosis with a 4,5–6,5 Fr Semi Rigid Ureterorenoscope, so a ureteroneocystostomy (Politano-Leadbetter ureteral reimplantation) was performed. In the same operating session, the ureteral stone was removed and a left orchidopexy was performed. After 3 months, a percutaneous nephrolithotripsy was performed to treat the staghorn stone; a nephrostomy was left and a subsequent anterograde pyelography showed no uretero-pelvic obstruction and a normal vesico-ureteral junction after reimplantation. Moreover, in both cases the analysis of the stone fragments revealed a 100% cystine composition.

The diagnosis of cystinuria was confirmed by urinary amino acid examination (cystine/creatinine 676 mMol/mol, normal value 4–15). Tiopronin (15 mg/kg/day) was immediately started, along with Potassium Citrate. Given the biochemical diagnosis of cystinuria and the clinical evidence of hypotonia, MLPA analysis for SLC3A1 and PREPL deletions was performed in both the patient and his parents.

The test identified SLC3A1:E7_10del/PREPL:E1-14 deletion and SLC3A1: E2_E10/PREPL:E4-E14 del in the father's and mother's proband, respectively.

The child inherited father's and mother's deletion in SLC3A1 and PREPL with the loss of exons 7–10 in SLC3A1 an 4–14 in PREPL gene.

After 1 year of treatment, the patient did not show any recurrence of kidney stones. Neurological work up showed an improvement in both hypotonia and in dysphagia with no need of oro-gastric tube after 24 months of life. The patent *foramen ovale* with left-right shunt and atrial septum aneurysm still persists (POF 8 mm) at the age of 24 months. Growth impairment is still present but slowly improving.

## Discussion

**Figure 1:**
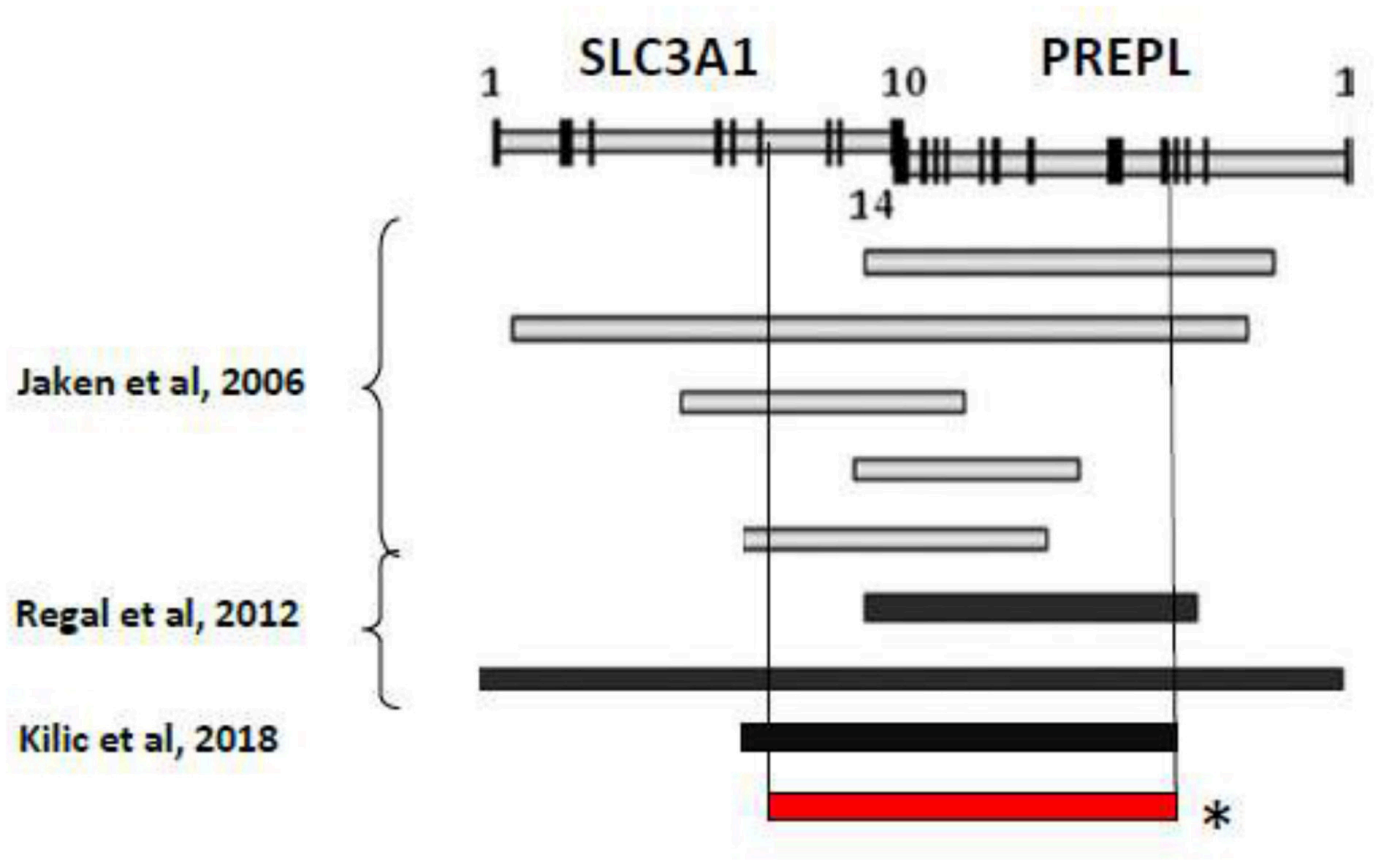
Summary of reported deletion in the 2p21 associated to HCS. ^*^Genetic mutation of the described patient.

Several autosomal recessive contiguous gene deletion syndromes have been described at 2p21 locus and the clinical spectrum depends on the size of the deletion and on the number of genes involved: 2p21 deletion syndrome (PPM1B, SLC3A1, PREPL, C2orf34) (5), HCS syndrome (SLC3A1, PREPL) (1, 6), atypical HCS (SLC3A1, PREPL, C2orf34) (7) and PREPL-C2orf34deletions (8) (Figure 1).

We here describe the case of a 2 years old child with HCS and a homozygous deletion in exons 7–10 of SLC3A1 and 4-14 of PREPL. The patient presents HCS with classic neuromuscular involvement and urolithiasis but also unusual clinical features. Indeed, the child presents a primary obstructive megaureter associated with unilateral cryptochidism with also cardiological involvement (patent *foramen ovale* with atrial septum aneurysm).

The pathogenesis of congenital anomalies of the kidney and urinary tract (CAKUT) is caused by the disturbance of normal nephrogenesis, secondary to environmental and genetic causes. The observation of kidney and urinary tract malformations as a feature of complex genetic syndromes has recently enlightened the contribution of genetics in CAKUT and is driving research efforts toward its understanding. So far, some genes have been identified as responsible of CAKUT phenotypes, being PAX2 and HNF1beta the most commonly identified (9). Nevertheless, in most cases the genetic basis of CAKUT phenotype remains unknown. CAKUT genes when mutated can be responsible of syndromic manifestations, but, so far, none of them has been described as associated to hypotonia and/or cystinuria.

Our patient presented monolateral cryporchidism and primary obstructive megaureter (POM) that have never been reported in patients with HCS. So far, only Bartholdi et al. (10) described a genitourinary involvement (with hypospadia and hypoplastic labia majora) as associated to PREPL deletion in 2 cases of 2p21 syndrome with atypical presentation. Among 26 HCS patients from 20 families with nine different deletions reported so far, only Kiliç et al. (11) have recently described the association between HCS and cardiac manifestation for the first time. The authors report a case of HCS associated with dilated cardiomiopathy in a 7-months-old female with a novel deletion of PREPL, which corresponds to our patient's PREPL deletion. PREPL is highly expressed in the brain and mildly expressed in the skeletal muscle, heart and kidney (6).

In this context, we cannot exclude that PREPL has a role in our patient's phenotype, being highly expressed not only in the central nervous system and in the striated muscle, but also in the genitourinary system and in the heart. Only whole exome sequencing can exclude other genes to be involved in the phenotype.

## Author Contributions

FT conceptualized the paper, drafted the initial manuscript and approved the final manuscript as submitted. VC conceptualized the paper, revised the initial draft, and approved the final manuscript as submitted. AB and ED revised the initial draft and approved the final manuscript as submitted. GAM and GM conceptualized the paper, revised the initial manuscript and approved the final manuscript as submitted.

### Conflict of Interest Statement

The authors declare that the research was conducted in the absence of any commercial or financial relationships that could be construed as a potential conflict of interest.

## Abbreviations

HCS: Hypotonia Cystinuria Syndrome
CAKUT: Congenital Anomalies of the Kidney and Urinary Tract
POM: Primary Obstructed megaureter
POF: patent *foramen ovale*.
